# The Value of Combined Detection of D-dimer and CD62p in Judging the Severity of Acute Cerebral Infarction and Short-Term Prognosis

**DOI:** 10.1155/2021/6620311

**Published:** 2021-01-14

**Authors:** Min Xu, Xiao-ying He, Pan Huang

**Affiliations:** ^1^Department of Neurology, The Second People's Hospital of Deyang City, No. 340 Minjiang West Road, Deyang, Sichuan 618000, China; ^2^Department of Neurology, The Affiliated Hospital of Southwest Medical University, No. 25 Taiping Street, Luzhou, Sichuan 646000, China; ^3^Department of Neurology, People's Hospital of Deyang City, No. 173 TaiShan North Road, Deyang, Sichuan 618000, China

## Abstract

**Objective:**

To explore the value of combined detection of peripheral blood P-selectin (CD62p) and D-dimer (D-dimer) in the judgment of acute cerebral infarction severity and short-term prognosis.

**Methods:**

268 patients with acute cerebral infarction from February 2015 to February 2019 were selected as the observation group. According to the National Institute of Health stroke scale, there were 90 cases (SCI group), 88 cases (MOCI group), and 90cases (MICI group) in the severe, moderate, and mild cerebral infarction groups, respectively. In the same period, 80 cases of healthy people served as the Normal group. Use flow cytometry to detect CD62p in peripheral blood and magnetic bead method to detect D-dimer level within 24 hours of onset. Logistic regression was used to analyze whether the two are factors affecting the short-term prognosis of acute cerebral infarction, and the ROC curve was drawn to evaluate the value of the combined detection of the two in the short-term prognosis of patients with acute cerebral infarction.

**Results:**

Peripheral blood D-dimer and CD62p levels (2.95 ± 0.76 ng/l, 34.03 ± 5.29 ng/l) in the SCI group were higher than those in the MOCI group (2.30 ± 0.51 ng/l, 27.58 ± 5.56 ng/l) and the MICI group (1.87 ± 0.40 ng/l, 19.60 ± 3.98 ng/l); the difference between the groups was statistically significant (*P* < 0.05). Logistic regression analysis showed that D-dimer and CD62p were independent risk factors affecting the poor prognosis of patients with acute cerebral infarction (OR values were 3.752 and 1.213, and 95% CI were 1.612-7.934 and 1.093-1.342, respectively, both *P* < 0.05). The AUC of D-dimer combined with CD62p for predicting poor prognosis of acute cerebral infarction is 0.859, which is better than D-dimer and CD62p alone.

**Conclusion:**

Peripheral blood D-dimer combined with CD62p detection is helpful for the risk stratification and short-term prognosis assessment of patients with acute cerebral infarction. Clinical detection is of great significance for the prevention and monitoring of disease development.

## 1. Introduction

Acute cerebral infarction (ACI) is a common clinical disease and frequently occurring disease. Due to the nonrenewable nature of neurons, cerebral infarction has a very high disability rate and recurrence rate, which brings a serious burden to society and families. Therefore, it is very important to predict the early stage of high-risk patients with cerebral infarction and actively rescue the ischemic penumbra in the early stage after cerebral infarction, restore the blood supply of brain cells to slow down the brain cell damage, and improve the clinical treatment rate of patients [1]. Studies have found that platelet activation, coagulation system, and fibrinolytic system imbalance are the main causes of cerebral thrombosis [2, 3]. P-selectin (CD62p), as a glycoprotein on the surface of platelets, can reflect the level of platelet activation, and D-dimer is a clear marker that reflects the high specificity of thrombosis and activation of the fibrinolytic system, which helps thrombosis monitoring of formation and thrombolytic therapy. In order to clarify the application value of the two in the severity of acute cerebral infarction and short-term prognosis, this study performed D-dimer and CD62p detection on 268 patients with acute cerebral infarction admitted to our hospital. The report is as follows.

## 2. Materials and Methods

### 2.1. Clinical Data

**Figure 1:**
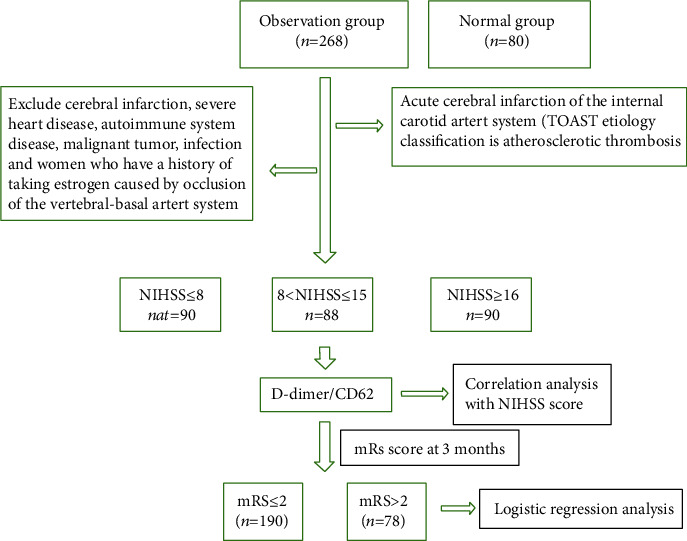
Flow chart.

268 patients with acute cerebral infarction who were hospitalized in the Department of Neurology of our hospital from February 2015 to February 2019 were selected as the cerebral infarction group, including 148 males and 120 females, aged 45-82 (68.78 ± 9.72) years old. All selected cases meet the diagnostic criteria for acute ischemic stroke [4]. According to the diagnosis results of hematology, imaging, and cardiac examination, TOAST classification was carried out and confirmed by two researchers for consistency and acute cerebral infarction of the internal carotid artery system (TOAST etiology classification is atherosclerotic thrombosis). Also, exclude posterior circulation cerebral infarction, chronic obstructive pulmonary disease, ischemic heart disease, rheumatic heart disease, congenital heart disease, bacterial endocarditis and atrial fibrillation, autoimmune system disease, malignant tumor, infection, severe medical diseases (such as pulmonary embolism, venous sinus thrombosis, deep vein thrombosis), and women who have a history of taking estrogen. After the patients were admitted to the hospital, the cerebral infarction group was divided into 3 subgroups according to the National Institute of Health stroke scale: [5] MICI group 90 cases (≤8 scores), MOCI group 88 cases (8-15 scores), and MICI group 90 cases (>16 scores). A total of 80 patients were selected as the Normal group during the outpatient health examination in this hospital, including 42 males and 38 females, aged 46-81 (63.85 ± 10.34) years old. This study was approved by the Ethics Committee of Deyang People's Hospital, ethics approval number 2014-11-02. The test flow chart is shown in Figure 1.

### 2.2. Specimen Collection and Measurement Methods

All subjects were taken fasting anterior elbow venous blood before receiving treatment after admission 3 ml. Among them, the EDTA anticoagulant tube was used to separate and extract the plasma, and the anticoagulant tube was not added to separate and extract the serum and stored in the refrigerator at -20°C. The CD62p level in serum was detected by flow cytometry (flow cytometer was American Applied Biosystems; the kit was Shanghai Xuanhao Technology Co., Ltd., operated according to the instructions). The content of D-dimer in plasma was detected by the magnetic bead method (normal value: 0-1 *μ*g/ml).

### 2.3. Prognosis Follow-Up

After 3 months of onset, follow-up by telephone or outpatient service, evaluate the functional prognosis of the patients according to the mRS score, and group them according to the prognosis of the patients. mRS score ≤ 2 is a good prognosis group, and mRS score > 2 is a poor prognosis group. Analyze the affected patient prognostic factors. The blind collection is used for data collection.

### 2.4. Statistical Methods

Using the SPSS17.0 statistical software, the measurement data is expressed as*x* ± *s*, the independent sample*t*-test is used for the comparison of the two groups, the ANOVA analysis is used for the comparison of multiple groups, and the LSD-t method is used for the pairwise comparison. Logistic regression was used to analyze whether D-dimer and CD62p are factors affecting the prognosis of patients with acute cerebral infarction, and the ROC curve was drawn to calculate the area under the curve (AUC). *P* < 0.05 indicates that the difference is statistically significant.

## 3. Result

### 3.1. Comparison of Baseline Data between the Observation Group and Control Group

There was no significant difference in the clinical data and laboratory examination between the observation group and the control group (*P* > 0.05) (Table 1).

### 3.2. The Levels of D-dimer and CD62p in the Peripheral Blood of the Observers Were Compared

The results showed that the levels of D-dimer and CD62p in the cerebral infarction group were higher than those in the normal group, and the difference was statistically significant (*P* < 0.05). The differences between the SCI group, MOCI group, and MICI group are also statistically significant (Table 2).

### 3.3. Correlation Analysis of D-dimer, CD62p Levels, and NIHSS Scores in the Observation Group

**Figure 2:**
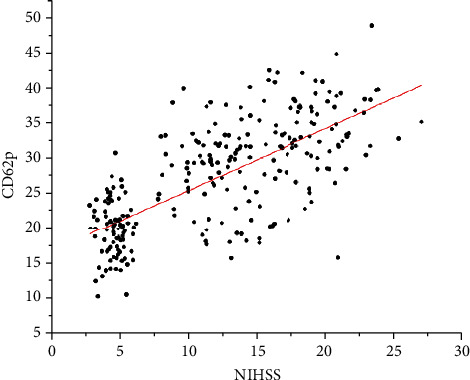
Correlation analysis between CD62p and NIHSS score.

**Figure 3:**
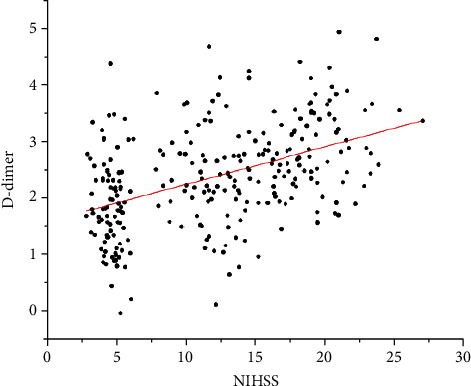
Correlation analysis between D-dimer and NIHSS score.

The results showed that D-dimer, CD62p, and NIHSS scores were all positively correlated (*r* = 0.455, 0.707; *P* < 0.05) (Table 3, Figures 2 and 3).

### 3.4. Factors Affecting the Prognosis of Patients with Acute Cerebral Infarction

WithmRS ≤ 2points in March as the good prognosis group (190 cases), mRS>2 points as the poor prognosis group (78 cases); D-dimer, CD62p, and general clinical data were used as independent variables for logistic regression analysis. It is shown that smoking history, D-dimer, and CD62p are all independent predictors of poor prognosis, as shown in Table 4.

### 3.5. The Predictive Value of D-dimer and CD62p on the Poor Prognosis of Acute Cerebral Infarction

**Figure 4:**
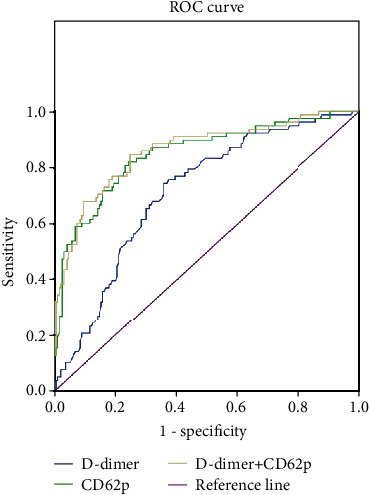
ROC curve.

The AUC of D-dimer and CD62p alone in predicting poor prognosis of acute cerebral infarction were 0.712 and 0.848 (*P* < 0.05), respectively. The combined detection of the two to predict the poor prognosis of acute cerebral infarction AUC was 0.859 (*P* < 0.05) (Table 5 and Figure 4).

## 4. Discussion

Acute cerebral infarction is a common clinical thromboembolic disease with an extremely high risk of disability, death, and recurrence. Therefore, it is of great significance to actively seek specific markers that can predict the severity and prognosis of the disease. Most of the TOAST etiological classification of cerebral infarction is atherosclerosis, and its pathological basis is platelets. Studies have found that platelet activation is closely related to the occurrence and development of thrombosis. Cerebral infarction is based on atherosclerotic plaques. Due to changes in hemodynamics or blood contents, platelet activation is intensified, forming platelet-vininogen thrombus, which blocks blood vessels and causes infarction [6–8].

Various reasons cause the damaged blood vessel wall to contract and change the local hemodynamic characteristics, which leads to the adhesion of platelets to the exposed subendothelial tissue under the action of plasma von Willebrand factor (VWF). The stimulation of collagen or thrombin released by endothelial cells undergoes morphological changes, and further release reaction and arachidonic acid metabolism occur. The ADP secreted by the release reaction and the metabolism of arachidonic acid to form thromboxane A2 can activate intracellular pathways to make Ca ion flow, which causes the bridging of platelets and fibrin to cause aggregation, and finally, plasma fibrinogen participates in the aggregation to form thrombus. The platelet adhesion-deformation-release-aggregation reaction in this process is the platelet activation reaction [9, 10]. It is precisely because platelet activation has such an important role; this study is based on this observation in order to find new ways to predict the severity and prognosis of the disease.

CD62p is a glycoprotein distributed on *α* particles in stationary platelets. When platelets are activated, *α* particles quickly fuse with the platelet membrane and release, so that CD62p is redistributed on the surface of platelets [11]. Studies have found that the concentration peak is reached 10 minutes after platelet activation, so it can be used as a specific marker for evaluating platelet activation status and thrombosis. Okada used mice and baboons to establish a model of cerebral ischemia and found that the expression of CD62p increased after 1 hour of cerebral ischemia, reaching a peak at 8 to 24 hours and lasting for 3 to 5 days [12]. The domestic scholar HUANG Tong dynamically detects CD62p in patients with acute cerebral infarction, and the results are basically consistent with OKada [13]. Cha observation of 45 patients with acute cerebral infarction showed that the upregulation of CD62p is related to the clinical deterioration of patients with acute cerebral infarction [14]. In addition, acute cerebral infarction is often associated with dysfunction or disorder of the fibrinolytic system. D-dimer is a specific degradation product of cross-linked fibrin, the increase of peripheral blood D-dimer indicates the enhancement of in vivo fibrinolytic activity. Studies have found that D-dimer will only increase in plasma after thrombosis in the body, so it can be used as a specific molecular marker for the diagnosis of thrombosis. Studies have shown that D-dimer is related to the onset and recurrence of coronary heart disease and is also related to cerebral venous sinus thrombosis [15, 16].

This study shows that the levels of CD62p and D-dimer in patients with acute cerebral infarction are significantly higher than those in the normal control group, indicating that the two can be used as markers of acute cerebral infarction thrombosis, which is consistent with other relevant experimental conclusions [17–19]. The levels of D-dimer and CD62p can be dynamically monitored during clinical thrombolysis to evaluate whether thrombus fusion is possible. The levels of D-dimer and CD62p in the SCI group were higher than those in the MOCI group and the MICI group, and the differences were statistically significant (*P* < 0.05), indicating that the higher the degree of platelet activation, the stronger the platelet adhesion and aggregation ability, and the easier to form arteries. Thrombus causes local brain tissue blood supply to be interrupted to form infarcts. Logistic regression analysis shows that D-dimer and CD62p are independent risk factors affecting the prognosis of patients with cerebral infarction. ROC curve analysis shows that peripheral blood D-dimer and CD62p levels have a higher predictive value for the poor prognosis of patients with acute cerebral infarction, which is consistent with the conclusions of other scholars [20, 21]. Therefore, the detection of peripheral blood D-dimer combined with CD62p in patients with acute cerebral infarction is timely, convenient, and economical and can early assess the risk stratification and prognosis of patients with acute cerebral infarction. However, the sample size of the cases collected in this study is small and cannot be monitored dynamically, which needs to be further improved.

## Figures and Tables

**Table 1:** Comparison of baseline data between observation group and control group.

Item	Observation group (*n* = 268)	Normal group (*n* = 80)	*P*
Age (years)	64.42 ± 8.72	63.97 ± 8.55	0.68
Sex (M/F)	136/132	38/32	0.85
Smoke (*n*, %)	145 (54.10%)	47 (58.75%)	0.76
Hypertension (*n*, %)	51 (19.02%)	15 (18.75%)	0.46
Diabetes (*n*, %)	41 (15.29%)	12 (15.00%)	0.09
SCr (*μ*mol/l)	52.23 ± 6.46	51.89 ± 6.21	0.67
UA (*μ*mol/l)	354.47 ± 37.15	361.12 ± 39.47	0.17
TC (mmol/l)	4.87 ± 0.42	4.91 ± 0.43	0.45
TG (mmol/l)	1.22 ± 0.24	1.27 ± 0.28	0.07
HDL-C (mmol/l)	0.93 ± 0.23	0.96 ± 0.24	0.61
LDL-C (mmol/l)	2.89 ± 0.43	2.83 ± 0.40	0.45
AST/ALT	0.75 ± 0.23	0.72 ± 0.22	0.65
BMI (kg/m^2^)	22.56 ± 1.23	22.74 ± 1.27	0.69
Drinker (*n*,%)	82 (30.59%)	22 (27.5%)	0.58

BMI: body mass index; TG: triglyceride; TC: total cholesterol; HDL-C: high-density lipoprotein-cholesterol; LDL-C: low high-density lipoprotein-cholesterol.

**Table 2:** Comparison of D-dimer and CD62p levels in peripheral blood of each group (*x* ± *s*).

Item	*n*	NIHSS scores	D-dimer (*μ*g/ml)	CD62p (ng/l)
Normal	80	0	0.87 ± 0.23	16.20 ± 3.26
SCI	90	4.60 + 0.76	1.87 ± 0.40^∗^	19.60 ± 3.98^∗^
MOCI	88	12.40 + 2.56	2.30 ± 0.51^#^	27.58 ± 5.56^#^
MICI	90	18.79 + 2.98	2.95 ± 0.76^∗^^#^	34.03 ± 5.29^∗^^#^
*P*			0.00	0.00

^∗^The MICI group compares with the normal group *P* < 0.05; ^#^The MOCI group compares with the MICI group *P* < 0.05; ∗^#^The SCI group compares with the MICI group *P* < 0.05.

**Table 3:** Correlation analysis of D-dimer, CD62p, and NIHSS score.

	NIHSS score	
	*r*	*P*
D-Dimer	0.455	0.00
CD62p	0.707	0.00

**Table 4:** Logistic regression analysis of factors affecting the prognosis of acute cerebral infarction.

Item	OR	95% CI	*P*
Age	1.052	0.883-1.256	0.556
Hypertension	7.281	0.458-113.230	0.152
Diabetes	4.352	0.426-44.684	0.214
Smoke	4.102	1.086-15.234	0.035
Drinke	0.715	0.064-8.231	0.724
SCr	0.269	0.023-3.487	0.312
BMI	1.213	0.115-12.485	0.885
D-Dimer	3.572	1.612-7.934	0.002
CD62p	1.213	1.093-1.342	0.003

**Table 5:** The predictive value of peripheral blood D-dimer and CD62p detection for end-point events.

Item	AUC	Standard error	95% CI	*P*
D-Dimer	0.712	0.033	0.647-0.777	0.026
CD62p	0.848	0.028	0.794-0.902	0.019
D-Dimer+CD62p	0.859	0.027	0.807-0.911	0.005

## Data Availability

All data, models, and code generated or used during the study appear in the submitted article.
